# Morbimortality in adult patients with septic arthritis: a three-year hospital-based study

**DOI:** 10.1186/s12879-016-1540-0

**Published:** 2016-06-01

**Authors:** Julien Ferrand, Youssef El Samad, Benoit Brunschweiler, Franck Grados, Nassima Dehamchia-Rehailia, Alice Séjourne, Jean-Luc Schmit, Antoine Gabrion, Patrice Fardellone, Julien Paccou

**Affiliations:** Department of Rheumatology, Amiens University Hospital, F-80054 Amiens, France; Department of Infectious Diseases, Amiens University Hospital, F-80054 Amiens, France; Department of Orthopaedics, Amiens University Hospital, F-80054 Amiens, France

**Keywords:** Septic arthritis, Clinical characteristics, Mortality, Poor functional outcome, Risk factors, C-reactive protein

## Abstract

**Background:**

The objective of this ambispective study was to determine outcomes and associated factors for adult patients with confirmed septic arthritis (SA).

**Methods:**

All adult patients admitted to Amiens University Hospital between November 2010 and December 2013 with confirmed SA were included in the study. Patients with prosthetic joint infections were excluded. A statistical analysis was performed in order to identify risk factors associated with a poor outcome (including mortality directly attributable to SA).

**Results:**

A total of 109 patients (mean ± SD age: 60.1 ± 20.1; 74 male/35 females) were diagnosed with SA during the study period. The most commonly involved sites were the small joints (*n* = 34, 31.2 %) and the knee (*n* = 25, 22.9 %). The most frequent concomitant conditions were cardiovascular disease (*n* = 45, 41.3 %) and rheumatic disease (*n* = 39, 35.8 %). One hundred patients (91.7 %) had a positive microbiological culture test, with S*taphylococcus aureus* as the most commonly detected pathogen (*n* = 59, 54.1 %). Mortality directly attributable to SA was relatively infrequent (*n* = 6, 5.6 %) and occurred soon after the onset of SA (median [range]: 24 days [1–42]). Major risk factors associated with death directly attributable to SA were older age (*p* = 0.023), high C-reactive protein levels (*p* = 0.002), diabetes mellitus (*p* = 0.028), rheumatoid arthritis and other inflammatory rheumatic diseases (*p* = 0.021), confusion on admission (*p* = 0.012), bacteraemia (*p* = 0.015), a low creatinine clearance rate (*p* = 0.009) and the presence of leg ulcers/eschars (*p* = 0.003). The median duration of follow-up (in patients who survived for more than 6 months) was 17 months [6–43]. The proportion of poor functional outcomes was high (31.8 %). Major risk factors associated with a poor functional outcome were older age (0.049), hip joint involvement (*p* = 0.003), the presence of leg ulcers/eschars (*p* = 0.012), longer time to presentation (0.034) and a low creatinine clearance rate (*p* = 0.013).

**Conclusions:**

In a university hospital setting, SA is still associated with high morbidity and mortality rates.

## Background

Septic arthritis (SA) is the most serious condition in the differential diagnosis of an inflamed, swollen joint [1–3]. The diagnosis of SA can be challenging even for doctors skilled in the management of musculoskeletal disease [1–3]. The incidence of SA in the USA and Western Europe has variously been reported as between 2 and 10 per 100,000 patient-years [4–7]. The incidence rises in specific patient populations; in patients with rheumatoid arthritis (RA), the annual incidence has been estimated at 70 cases per 100,000 patient-years [8, 9]. Additional risk factors for SA include low socioeconomic status, previous rheumatic disease (e.g. RA, osteoarthritis, crystal arthropathy, and other forms of inflammatory arthritis), previous surgery, diabetes mellitus, leg ulcers, intravenous drug abuse, alcohol abuse, intra-articular corticosteroid injection, and (possibly) human immunodeficiency virus infection [10–12]. Although all age groups can be affected, SA mainly usually arises in elderly people and very young children [1–3]. In all age and risk groups, the most frequent causative organism is *Staphylococcus aureus*, followed by other Gram-positive bacteria (including streptococci) [1–3].

Septic arthritis is a medical emergency, and so delayed or inadequate treatment can lead to irreversible joint destruction and thus substantial morbidity and mortality [1–3]. In the literature, the reported mortality rate for SA ranges from 4 to 42 % [13–15], with values as high as 50 % in polyarticular disease [12]. Morbidity is also substantial, with persistent joint dysfunction occurring in up to 30 % of cases [16, 17]. However, very few detailed studies of morbidity, mortality and outcomes in adult patients with SA have been published in the last 20 years. The primary objective of the present study was therefore to identify (i) characteristics and outcomes in adult patients with SA admitted to a university hospital, and (ii) the risk factors associated with poor outcomes (including mortality).

## Methods

### Design and setting

This hospital-based, ambispective study was conducted at Amiens University Hospital (Amiens, France). Episodes of SA in adult patients (aged ≥16 years) attending this tertiary hospital over a three-year period (November 2010 to December 2013) were identified. Patients with prosthetic joint SA or patients with SA following surgical procedures were excluded from the study, as were patients with diabetes-related foot infections. The study was designed in accordance with the ethical standards of our hospital Ethics Committee (Amiens University Hospital). In view of the study’s design, no written informed consent to participation was necessary in accordance with our ethical committee. Indeed, this is an observational study with usual care.

### Case definition

All included patients met one of Newman’s diagnostic criteria for SA [18]: (A) isolation of an organism from an affected joint, (B) isolation of an organism from another site in a patient with a concomitant swollen, inflamed joint, (C) no organism isolated but clinical features and turbid joint fluid in patients having undergone prior antibiotic therapy, and (D) no organism isolated but histological or radiologic evidence consistent with SA.

Moreover, organisms were isolated and identified with conventional biochemical assays and tests in Amiens University Hospital’s microbiology laboratory.

### Case identification and data collection

The following methods were applied to identify cases: **(i)** consecutive patients with SA diagnosed between November 2010 and December 2013 were prospectively recorded in the diagnosis registry by the attending clinicians (in the rheumatology, orthopaedics and infectious disease departments); **(ii)** patients were identified retrospectively (in January 2014) by checking the hospital activity index (HAI) over a three-year period (November 2010–December 2013).

The patients’ case notes were reviewed from January 2014 onwards by using a structured *pro forma* report form. The following items of information were extracted: age, gender, concomitant conditions, presentation, assessments, investigations, pathogen(s) responsible, treatments and outcomes. If several joints were involved, the patient was classified as a function of the predominantly affected joint.

### Data on disease outcomes

Data on disease outcomes (collected between January 2014 and July 2014) were obtained from medical records and, if necessary, from the patients in a phone interview. When patients could not be contacted or were unable to answer questions, a family member or general practitioner was interviewed instead. The disease outcomes questionnaire included a number of closed questions: “Is the patient still alive?”; “When did death occur?”; “What was the cause of death?”; “Has your joint function worsened or have you developed any new limitations in joint function that can be ascribed to SA”.

After a review of the medical records (and a phone interview, if necessary), the *patient outcome* was classified as “poor” in the event of death. We sought to distinguish between deaths directly attributable to septic arthritis and those due to other causes. We considered that the death was related to SA when this was indicated in the patient’s case notes. The *joint outcome* was classified as “poor” in cases of amputation, arthrodesis, prosthetic surgery or severe functional deterioration reported by the patient. If several joints were involved, the patient was analyzed as a function of the predominantly affected joint.

### Statistical analysis

Statistical analysis was performed using SAS software (version 9.3, SAS Institute Inc., Cary NC, USA). Continuous variables are reported as the median, minimum, maximum, and missing values; categorical variables are reported as the frequency and percentage. All tests were two-sided, with a significance threshold set to *p* < 0.05. The various groups were compared using a chi-square test or Fisher’s exact test for categorical variables, and either an analysis of variance (or Student’s *t* test, for two groups) or a Kruskal–Wallis test (or a Mann–Whitney test, for two groups) for continuous variables.

A univariate logistic regression model was used to screen for risk factors that were predictive of mortality attributable directly to SA. Likewise, a univariate logistic regression model was used to screen for risk factors that were predictive of a poor functional outcome.

The following potential risk factors were tested: age, confusion on admission, time to presentation (days), open and arthroscopic surgery, diabetes mellitus, cardiovascular disease, cancer (pre-existing or concomitant), concomitant or pre-existing skin involvement (eschars and/or leg ulcers), previous RA and other forms of inflammatory arthritis, joint involvement (hip, knee, multiple or small joints), organism (*Staphylococcus aureus*), bacteraemia, C-reactive protein (CRP) and the measured creatinine clearance rate (evaluated according to the Modification of Diet in Renal Disease equation).

## Results

### Identification and selection of cases

**Fig. 1:**
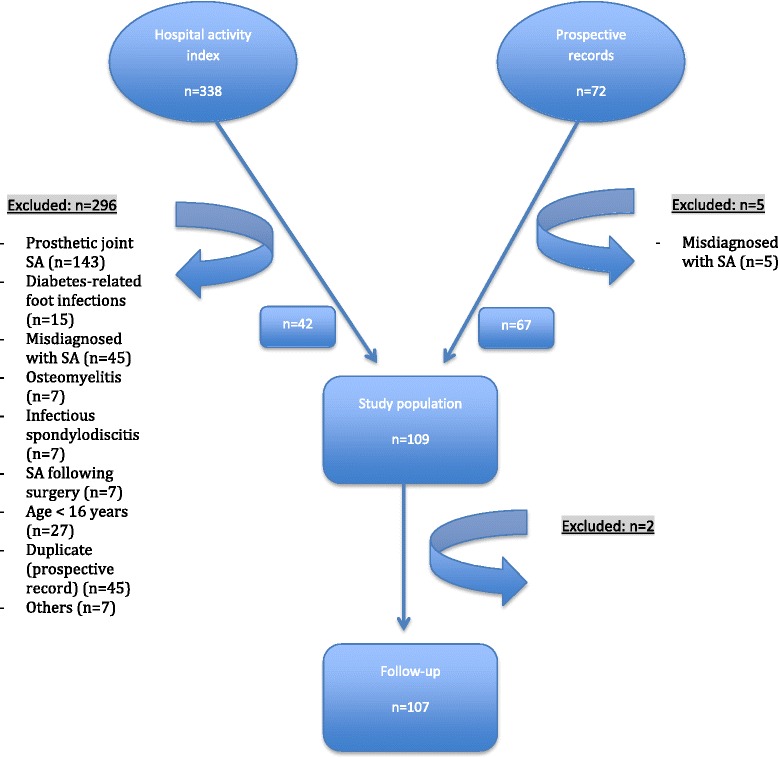
Study flow chart

A total of 410 adult patients were identified for the study period (Fig. 1). Twenty-two of the 410 patients were identified in the prospective diagnosis registry but not in the retrospective review. Two hundred and ninety-six patients were excluded following review of their case notes: 143 had prosthetic joint SA, 27 were aged under 16, 15 had diabetes-related foot infections, 7 had developed SA following surgery, 7 had osteomyelitis, 7 had infectious spondylodiscitis, 45 were duplicate cases (prospective records) and 45 had been misdiagnosed with SA (cases of RA, reactive arthritis, gout, etc.). Hence, data on the remaining 109 adult patients with confirmed SA (including 11 with multiple joint involvements) were analyzed. Most of the patients had been referred to our rheumatology (*n* = 51, 47.8 %) and orthopaedics departments (*n* = 37, 33.9 %), with fewer referrals to our infectious disease (*n* = 20, 18.3 %) and geriatric medicine departments (*n* = 1, <1 %) (Table 1).

**Table 1:** Characteristics of the study participants

Variables	Number	All participants (*n* = 109)	Small joint involvement (*n* = 34)	Large joint involvement (*n* = 75)	p-value
Age, years	109	62 [16–96]	52.5 [16–89]	65 [17–96]	**<0.01**
Male gender, n (%)	109	74 (67.9)	19 (55.9)	55 (73.3)	0.07
Body mass index (kg/m^2^)	80	25 [14.5–48]	24.1 [17–40.4]	26.05 [14.5–48]	0.25
Creatinine clearance (ml/min)	105	80 [5–150]	84 [13–150]	78 [5–150]	0.35
C-reactive protein (mg/l)	102	120 [3–640]	23 [3–230]	166 [3–640]	**<0.01**
Length of hospital stay (days)	109	16 [2–216]	6 [2–56]	21 [2–216]	**<0.01**
Duration of oral therapy (days)	102	52.5 [3–112]	42 [3–84]	84 [10–112]	**<0.01**
Department, n (%)	109				
-Rheumatology		51 (47.8)	5 (14.7)	46 (61.4)	
-Orthopaedics		37 (33.9)	28 (82.3)	9 (12.0)	
-Infectious Dis.		20 (18.3)	1 (3.0)	19 (25.3)	
-Geriatric Med.		1 (<1)	0 (<1)	1 (1.3)	**<0.01**
*Staphylococcus aureus*, n (%)	109	59 (59.0)	16 (51.6)	43 (62.3)	0.31
Diabetes, n (%)	109	26 (23.8)	4 (11.8)	22 (29.3)	**0.04**
Cardiovascular disease, n (%)	109	45 (41.3)	6 (17.6)	39 (52.0)	**<0.01**
Cancer, n (%)	109	22 (20.2)	4 (11.8)	18 (24.0)	0.14
Surgery, n (%)	109	60 (55.0)	26 (75.5)	34 (45.3)	**<0.01**
All-cause death, n (%)	107	19 (17.8)	2 (6.2)	17 (22.7)	**0.04**
Death directly attributable to SA, n (%)	107	6 (5.6)	0 (<1)	6 (8.0)	0.10
Poor functional outcome, n (%)	88	28 (31.8)	7 (23.3)	21 (36.2)	0.22

Data are reported as the median [range]. Statistically significant results are indicated in bold type

### Demographics, affected joints and pathogens

As mentioned above, 109 patients (with a total of 130 infected joints) were included (median [range] age: 62 [16–96]; *n* = 74 males, 67.9 %; median [range] BMI: 25 [14.5–48] kg/m^2^) (Table 1). In terms of the Newman criteria, 91 of the 109 patients (83.5 %) were in group A, with 9 in group B, 5 in group C and 4 in group D. Joint involvement is summarized in Table 2. The most frequently involved sited were the small joints (fingers and toes) (*n* = 34, 31.2 %), followed by the knee (*n* = 25, 22.9 %), the shoulder (*n* = 16, 14.7 %) and the hip (*n* = 12, 11.0 %). Patients with small joint involvement differed significantly in terms of age (52.0 ± 22.6 vs. 63.3 ± 18.4, *p* < 0.01) and most of them were referred to the orthopaedic department (*n* = 28, 82.3 %) (Table 1).

**Table 2:** Joint sites involved with SA (109 adult patients with confirmed SA (including 11 with multiple joint involvements with a total of 130 infected joints))

Joint involved	Number (*n* = 109)	Percent	Number (*n* = 130)	Percent
Small joint involvement (fingers and toes)	34	31.2	36	27.7
Knee	25	22.9	31	23.9
Shoulder (glenohumeral or acromioclavicular)	16	14.7	20	15.4
Hip	12	11.0	16	12.3
Ankle or subtalar	9	8.2	14	10.8
Sacroiliac	5	4.6	5	3.8
Symphisis pubis	3	2.7	3	2.3
Wrist	2	1.9	2	1.5
Elbow	2	1.9	2	1.5
Sternoclavicular	1	0.9	1	0.8

The data on bacterial isolates are summarized in Table 3. *Staphylococcus aureus* was the most commonly isolated bacteria (*n* = 54). Nine of the latter cases were due to methicillin-resistant strains. Streptococci and *Pasteurella* were involved in 11 and 7 episodes of SA, respectively.

**Table 3:** Organisms identified as causes of SA

Organism	Number (*n* = 109)
*Staphylococcus aureus*	*54*
*Staphylococcus* coagulase-negative	*5*
*Streptococcus*	*11*
*-* β-haemolytic streptococci	*7*
*- S. pneumoniae*	*2*
*-* Other streptococci	*2*
*Enterococcus*	*1*
*Pasteurella*	*7*
Coliform bacilli	*6*
*Neisseria gonorrhoeae*	*3*
Other	*4*
Mixed infections	*9*
Unknown	*9*

### Concomitant conditions

The most frequent concomitant conditions in our study population are listed in Table 4: cardiovascular disease (*n* = 45, 41.3 %), including heart disease (*n* = 37, 33.9 %) and peripheral arterial disease (*n* = 19, 17.4 %); pre-existing rheumatic disease (*n* = 39, 35.8 %), including osteoarthritis (*n* = 24, 22.0 %), gout/chondrocalcinosis (*n* = 15, 13.8 %), RA and other inflammatory rheumatic diseases (*n* = 7, 6.4 %). Furthermore, 6 of the 7 latter patients were taking immunosuppressants. Diabetes mellitus and cancer were noted in 26 (23.9 %) and 22 (20.2 %) patients, respectively. Concomitant or pre-existing skin involvement (eschars and/or leg ulcers) was found in 16 (14.7 %) patients. Patients with small joint involvement differed significantly from patients with large joint involvement in terms of cardiovascular disease (17.6 % vs. 52.0 %, respectively; *p* < 0.01) and diabetes (11.8 % vs. 29.3 %, *p* = 0.04) (Table 1).

**Table 4:** Concomitant conditions

Concomitant conditions	Number	Percent
Heart disease	37	33.9
Current smokers	36	33.0
Diabetes mellitus	26	23.9
Osteoarthritis	24	22.0
Previous cancer	22	20.2
Peripheral arterial disease	19	17.4
Kidney disease	17	13.8
Gout/chondrocalcinosis	15	13.8
Alcohol abuse	13	11.9
Lung disease	12	11.0
Oral corticosteroids	10	9.2
Skin ulceration	10	9.2
Eschars	9	8.2
Liver disease	7	6.4
Rheumatoid arthritis and other inflammatory rheumatic disease	7	6.4
Intra-articular corticosteroids	3	2.8
Intravenous drug abuse	2	1.8

### Diagnosis and presentation

The median [range] time to presentation was 7 [1–120] days. A history of fever was recorded in 44 % of cases, with rigor in 27 % and sweating in only 16 %. Fever (>37.5 °C) and confusion on admission were respectively detected in only about 46 % (48 of 104) and 12 % (13 of 109) of cases.

The CRP level was elevated (>3.2 mg/l) in 96.1 % of the 102 tested patients (median [range]: 120 [3–640] mg/l). Moreover, CRP levels were lower in patients with small joint involvement than in those with large joint involvement (23 [3–230] mg/l vs. 166 [3–640] mg/l, respectively, *p* < 0.01). Serum procalcitonin levels were rarely determined (in fewer than 30 % of patients). The median creatinine clearance rate was 80 [5–150] ml/min in the 105 tested patients, with variously <15 ml/min (*n* = 4), 15–29 ml/min (*n* = 7), 30–59 ml/min (*n* = 17), 60–89 ml/min (*n* = 34) and ≥90 ml/min (*n* = 43) (Table 1). The total white cell count was elevated (>10,000) in only 50 % of the 105 tested patients. Anaemia (haemoglobin <12 g/dl) was observed in 50 % of the 105 tested patients.

One hundred and nine patients had at least one sample sent for microbiological testing. One hundred of these (91.7 %) had a positive culture. Specifically, organisms were identified in the synovial fluid in 86 (84.3 %) of the 102 patients that had synovial fluid analysed. Of the 79 patients who had blood cultures, 31 (39.2 %) were positive. In 11 of these, blood cultures were the only positive result. Forty three patients had synovial fluid sent in blood culture media and 30 (69.8 %) were positive. In just one case, this was the only positive microbiological test. Lastly, 16S rRNA gene sequencing was the only positive microbiological test in 2 patients (revealing *Streptococcus agalactiae* and *Streptobacillus moniliformis*, respectively).

Transoesophageal echocardiography and transthoracic echocardiography were performed in 17 and 48 cases, respectively, and enabled the detection of four cases of infectious endocarditis.

### Treatment

All patients were hospitalized. The median length of hospital stay (LOS) was 16 [2–216] days. The duration of treatment was very variable, with median durations of intravenous therapy and oral therapy of 8 [2–31] days and 52.5 [3–112] days, respectively. It is noteworthy that initial intravenous therapy was administered in 95 of the 109 cases (87.1 %). Interestingly, patients with small joint involvement had a significantly shorter LOS (6 [2–56], vs. 21 [2–216] days for patients with large joint involvement; *p* < 0.01) and duration of oral therapy (42 [3–84] vs. 84 [10–112], respectively; *p* < 0.01) (Table 1). Various antibiotic combinations were used in initial intravenous treatment, with the most common being aminoglycoside and oxacillin (*n* = 34), aminoglycoside and vancomycin (*n* = 10), aminoglycoside and amoxicillin (*n* = 9) and aminoglycoside and amoxicillin/clavulanic acid (*n* = 8). In terms of oral therapy, the most commonly used combinations were rifampicin and fluoroquinolones (*n* = 35), followed by dalacin and fluoroquinolones (*n* = 15). Sixty of the 109 patients (55 %) required surgical lavage (arthroscopic washout in 8 cases and arthrotomy/washout in 52 cases).

### Disease outcomes

The disease outcomes data for two of the 109 patients (<2 %) were incomplete because the patients could not be contacted. Both of these cases (a 17-year-old male and a 60-year-old female) involved finger joint infection with *S. aureus*. The median duration of follow-up (in patients surviving for more than 6 months) was 17 months [6–43].

Nineteen of the patients with SA (17.8 %) died; 6 of these mortalities were considered to be directly attributable to SA (5.6 %) and occurred soon after the onset of the SA (median: 24 days [1–42]). The remaining 13 patients died from causes other than SA: cardiovascular disease (*n* = 6), bed confinement (*n* = 3), kidney failure (*n* = 1), macrophage activation syndrome (*n* = 1), suicide (*n* = 1) and cancer (*n* = 1).

Our univariate analysis suggested that older age (odds ratio (OR) [95 % confidence interval] per year: 1.09 [1.02–1.19]; *p* = 0.023), CRP (OR per unit: 1.01 [1.006–1.02]; *p* = 0.002), bacteraemia (OR 15.20 [2.31–298.70]; *p* = 0.015), skin involvement (OR: 14.83 [2.62–115.71]; *p* = 0.003), RA or other inflammatory rheumatic diseases (OR 9.60 [1.14–64.04]; *p* = 0.021), diabetes mellitus (OR 7.18 [1.31–54.31]; *p* = 0.028), confusion on admission (OR 9.10 [1.51–55.39]; *p* = 0.012) and a low creatinine clearance rate (OR per unit: 0.96 [0.92–0.99]; *p* = 0.009) were predictive of mortality directly attributable to SA (Table 5).

**Table 5:** Univariate analysis of factors possibly associated with mortality attributable directly to septic arthritis

	Yes (*N* = 6)	No (*N* = 101)	p-value	Odds ratio [95 % CI]	Univariate analysis *p-value*
**Age (years)**	**82.5 [59–96]**	**62.0 [16–95]**	**0.017**	**1.09 [1.02–1.19]**	**0.023**
Gender (Female)	3 (50 %)	31 (31 %)	0.380	2.26 [0.40–12.80]	0.335
Knee involvement	3 (50 %)	22 (22 %)	0.138	3.59 [0.63–20.61]	0.133
Hip involvement	0 (<1 %)	12 (12 %)	1	not defined	0.993
Multiple joint involvement	2 (33 %)	8 (8 %)	0.097	5.81 [0.73–35.11]	0.062
Cardiovascular disease	3 (50 %)	42 (42 %)	0.694	1.40 [0.25–7.91]	0.686
**Skin involvement**	**4 (67 %)**	**12 (12 %)**	**0.004**	**14.83 [2.62–115.71]**	**0.003**
**Rheumatoid arthritis and related conditions**	**2 (33 %)**	**5 (5 %)**	**0.049**	**9.60 [1.14–64.04]**	**0.021**
Previous cancer	3 (50 %)	19 (19 %)	0.100	4.32 [0.75–24.95]	0.087
**Diabetes mellitus**	**4 (67 %)**	**22 (22 %)**	**0.030**	**7.18 [1.31–54.31]**	**0.028**
Time to presentation (days)	11 [4–24]	7 [0–120]	0.695	0.98 [0.92–1.02]	0.507
**Confusion on admission**	**3 (50 %)**	**10 (10 %)**	**0.023**	**9.10 [1.51–55.39]**	**0.012**
**Creatinine clearance (MDRD, ml/min)**	**32 [23–66]**	**81 [5–150]**	**0.003**	**0.96 [0.92–0.99]**	**0.009**
**C-reactive protein (mg/l)**	**375 [88–640]**	**120 [3–465]**	**0.003**	**1.01 [1.006–1.02]**	**0.002**
*Staphylococcus aureus*	4 (67 %)	53 (58 %)	1	1.47 [0.27–11]	0.665
Joint surgery	1 (17 %)	57 (56 %)	0.091	0.15 [0.01–1.002]	0.093
**Bacteraemia**	**5 (83 %)**	**25 (25 %)**	**0.006**	**15.20 [2.31–298.70]**	**0.015**
Small joint involvement	0 (<1 %)	32 (32 %)	0.175	*not defined*	0.993

*MDRD* Modification of Diet in Renal Disease

Statistically significant results are indicated in bold type

The morbidity was also considerable, since a poor functional outcome was observed in 28 of the 88 eligible patients (31.8 %): eleven patients had severe functional deterioration; amputation was necessary in 8 patients, osteosynthesis in 4 patients and joint replacement in 5 patients. In terms of the hip joint, a poor functional outcome was found in 8 of the 10 eligible patients with severe functional deterioration in 5 patients and joint replacement in 3 patients. Data on univariate risk factors for poor functional outcome are summarized in Table 6. The analysis suggested that older age (OR per year: 1.02 [1.001–1.05]; *p* = 0.049), hip joint involvement (OR: 11.60 [2.65–81.29], *p* = 0.003), skin involvement (OR: 6.33 [1.60–31.55]; *p* = 0.012), time to presentation (OR per day: 1.02 [1.002–1.04], *p* = 0.034) and a low creatinine clearance rate (OR per unit: 0.98 [0.97–0.99]; *p* = 0.013) were associated with a poor functional outcome.

**Table 6:** Univariate analysis of factors possibly associated with a poor functional outcome in septic arthritis

	Yes (*N* = 28)	No (*N* = 60)	p-value	Odds ratio [95 % CI]	Univariate analysis *p-value*
**Age (years)**	64.0 [36–90]	54.5 [16–95]	0.076	**1.02 [1.001–1.05]**	**0.049**
Gender (Female)	10 (36 %)	19 (32 %)	0.707	1.20 [0.46–3.07]	0.707
Knee involvement	3 (11 %)	13 (22 %)	0.252	0.43 [0.09–1.50]	0.224
**Hip involvement**	**8 (29 %)**	**2 (3 %)**	**0.001**	**11.60 [2.65–81.29]**	**0.003**
Multiple joint involvement	3 (11 %)	4 (7 %)	0.675	1.68 [0.31–8.17]	0.517
Small joint involvement	7 (25 %)	23 (38 %)	0.219	0.54 [0.19–1.41]	0.223
Cardiovascular disease	12 (43 %)	19 (32 %)	0.306	1.62 [0.64–4.10]	0.308
**Skin involvement**	**7 (25 %)**	**3 (5 %)**	**0.010**	**6.33 [1.60–31.55]**	**0.012**
Rheumatoid arthritis and related conditions	1 (4 %)	3 (5 %)	1	0.70 [0.03–5.79]	0.765
Previous cancer	8 (29 %)	7 (12 %)	0.049	3.03 [0.97–9.73]	0.056
Diabetes mellitus	7 (25 %)	13 (22 %)	0.728	1.21 [0.40–3.40]	0.728
**Time to presentation (days)**	**15 [0–105]**	**5 [0–91]**	**0.016**	**1.02 [1.002–1.04]**	**0.034**
**Creatinine clearance (MDRD, ml/min)**	**71 [5–130]**	**94 [29–150]**	**0.019**	**0.98 [0.97–0.99]**	**0.013**
C-reactive protein (mg/l)	120 [3–342]	105.5 [3–465]	0.862	0.999 [0.995–1.004]	0.839
*Staphylococcus aureus*	15 (58 %)	29 (55 %)	0.802	1.13 [0.44–2.96]	0.803
Joint surgery	17 (61 %)	33 (55 %)	0.614	1.26 [0.51–3.21]	0.615

*MDRD* Modification of Diet in Renal Disease

Statistically significant results are indicated in bold type

## Discussion

This study of adult patients with SA specifically included only the 109 cases with native joint infection admitted to our university hospital. The duration of follow-up enabled us to determine the outcomes (including mortality directly attributable to SA). The frequency of death directly attributable to SA (5.6 %) was lower and the poor functional outcome rate (31.8 %) was higher than expected. Predictive factors of death directly attributable to SA were older age, high serum CRP levels, RA and other inflammatory disease, diabetes mellitus, and confusion on admission, bacteriema, skin involvement (leg ulcers and/or eschars) and a low creatinine clearance rate. Older age, hip joint involvement, skin involvement, a longer time to presentation and a low creatinine clearance rate were predictive of a poor functional outcome.

In terms of identifying individual cases, complete reliance on retrospective HAI data would have been a problem. Fortunately, consecutive patients with SA diagnosed between November 2010 and December 2013 were reported to a prospective diagnosis registry by our clinicians. Accordingly, 22 patients (20 % of the total) were identified solely through the diagnosis registry. Children, adolescents and patients with prosthetic joint SA were excluded from the study because we considered that (in comparison with adult patients with native SA) pathogens, treatments, outcomes, and risk factors for the development of SA might differ [14, 19–23].

Our results confirmed literature data on the demographics (age and gender) of patients with SA [12, 24], the affected joints, the pathogens and the risk factors. Surprisingly, the most common sites involved were small joints (31.2 %, which is much higher than the literature values of below 10 % [5, 6]). This disparity might be related to our inclusion criteria, since SA as a result of a skin wound is sometimes excluded from studies in this field; in the present study, this condition was frequently observed in patients with small joint involvement. Our results again emphasize that multiple joint involvement is not rare [1]. Indeed, 11 of our 109 patients had several affected joints. The spectrum of causative organisms was similar to that reported in previous studies, with the Gram-positive organisms *S. aureus* and *Streptococci* being responsible for 60 % of cases [6]. Septic arthritis due to MRSA was rare during the present study period (9 cases, accounting for 8.3 % of all episodes) [25]. We found that a high proportion (35.8 %) of patients with SA had underlying rheumatic disease, as reported previously [10, 12]. However, few patients (relative to the literature data) were affected by RA (*n* = 4) or other inflammatory rheumatic diseases (*n* = 3) [5, 12]. Surprisingly, pre-existing or concomitant cancer was reported in 22 patients. These conditions are rarely reported on in this kind of study. Intra-articular corticosteroid injection and human immunodeficiency virus infection were also quite rare [7, 10, 11].

Our data strongly suggested that a sample of synovial fluid should be obtained before antibiotic treatment is initiated. However, it is also clear that blood cultures contribute to the diagnosis and should not be omitted. Cultures of joint aspirate and/or blood were positive in 89 % of cases; this proportion is similar to that observed in previous studies [6, 26]. The aetiology of SA could not be determined in only 9 cases (8.2 %). Moreover, the use of blood culture methods for synovial fluids might not be of value; further studies are needed to unambiguously resolve this issue.

In the literature, mortality rates range from 4 to 42 % [15]. This broad range may be due to study-to-study difference in patient comorbidities, inclusion criteria (children, prosthetic joint infections, etc.) and clinical presentations (since SA can present as an isolated disease or as part of a sepsis syndrome. Two series published between 1997 and 1999 reported mortality rates of 11.5 and 10 %, respectively [5, 6], and Gupta et al. reported a value of 11 % in 2001 [12]. One series found a rate of 4 % [24] which is quite similar to our value of 5.6 %. More recently, Munoz-Egea et al. [27] and Lim et al. [28] reported rates of 7.7 % and 5.5 %, respectively. Surprisingly, Geirsson et al. found a mortality rate of 1.8 % in a study of 253 cases (69 children and 184 adults) diagnosed in Iceland between 1990 and 2002; an iatrogenic aetiology was noted in 41.8 % of the adults [7]. One possible explanation for the low mortality rate encountered in the present study is our decision to exclude prosthesis infections, since mortality rates for these patients are usually closer to 20 % [5]. Another possible explanation relates to the large number of cases of small-joint infections (fingers and toes), which generally correspond to limited-scale disease not accompanied by sepsis syndrome. Indeed, CRP levels were higher in patients with large joint involvement than in those with small joint involvement, and an elevated CRP level was a strong risk factor for deaths directly attributable to SA. To the best of our knowledge, our results are the first to highlight CRP in this respect. Although CRP *per se* was not tested as a predictive factor in previous studies [5, 6] an elevated erythrocyte sedimentation rate was predictive of death [12]. We do not have an obvious explanation for the predictive value of skin involvement (leg ulcers and/or eschars), although Gupta et al. [12] and Ravindran et al. [24] also found a higher rate of mortality in patients with leg ulcers. Again, the presence of eschars (whether previous or concomitant) had never previously been tested as predictive factor for death in SA. However, leg ulcers and above all eschars are found in the frailest patients; this perhaps explains why these features are associated with a high mortality rate. We also confirmed that older age, diabetes mellitus, bacteraemia, chronic renal failure, inflammation [29] and confusion [6] on admission are risk factors associated with death directly attributable to AS.

Furthermore, we observed substantial morbidity; a poor functional outcome occurred in 32 % of the patients (in line with the literature data) [5, 6, 24]. In the present study, older age, hip joint involvement, longer time to presentation, previous or concomitant skin involvement and a low creatinine clearance rate were associated with functional impairment. High morbidity in patients with SA at the hip joint can be explained by the frequent need for resection arthroplasty. We do not have obvious explanations for the correlation with skin involvement and a low creatinine clearance rate, although both conditions are associated with frailty (which probably prevented adequate recovery and rehabilitation). Longer time to presentation was also highlighted as a risk factor in the present study. This is particularly relevant, and physicians should be aware of this aspect. Moreover, the suspicion that surgical lavage may be associated with a poor outcome was not confirmed by our present results [6, 19].

Our study had several limitations. Firstly, the study was hospital-based (rather than population-based) and so its findings cannot be extrapolated to other populations. Secondly, our study had a relatively small number of patients. However, SA is a rare condition, and the implementation of multicentre, prospective, randomized controlled trials (to ensure large number of patients) is logistically difficult (albeit highly desirable). Thirdly, our study involved an unusually high proportion of adults with small joint involvement. However, small joint involvement emphasizes the broad spectrum of conditions in SA, since it can present as an isolated disease or as part of a sepsis syndrome. Lastly, the study was ambispective (rather than prospective), which prevented us from obtaining additional data (such as the patients’ functional status on admission, social status and education level).

## Conclusions

The present study emphasises the continuing poor outcomes following native SA in adults. Better patient outcomes will require additional research into the optimal management of SA (improved diagnosis, joint drainage methods, antibiotic regimens, treatment duration, etc.).

### Key messages

Septic arthritis continues to be associated with a considerable degree of morbidity and mortality.

C-reactive protein might a valuable predictive factor for death directly attributable to SA.

Hip joint involvement is associated with a poor functional outcome.

### Ethics approval and consent to participate

The study was designed in accordance with the ethical standards of our hospital Ethics Committee (Amiens University Hospital). In view of the study’s design, no written informed consent to participation was necessary in accordance with our ethical committee.

### Availability of data and materials

Data supporting our findings can be asked to Paccou Julien (julienpaccou@yahoo.fr)

## Abbreviations

BMI: body mass index
CRP: C-reactive protein
HAI: hospital activity index
LOS: length of hospital stay
MRSA: methicillin-resistant staphylococcus aureus
OR: odds ratio
RA: rheumatoid arthritis
SA: septic arthritis
